# Silver Nanoparticles Biosynthesized with Spruce Bark Extract—A Molecular Aggregate with Antifungal Activity against *Candida* Species

**DOI:** 10.3390/antibiotics10101261

**Published:** 2021-10-17

**Authors:** Anca Delia Mare, Adrian Man, Cristina Nicoleta Ciurea, Felicia Toma, Anca Cighir, Mihai Mareș, Lavinia Berța, Corneliu Tanase

**Affiliations:** 1 Department of Microbiology, “George Emil Palade” University of Medicine, Pharmacy, Sciences and Technology of Târgu Mureș, 38 Gheorghe Marinescu Street, Mureș, 540139 Târgu Mureș, Romania; anca.mare@umfst.ro (A.D.M.); adrian.man@umfst.ro (A.M.); anca.cighir@umfst.ro (A.C.); 2 Doctoral School, George Emil Palade University of Medicine, Pharmacy, Science and Technology of Târgu Mureș, 38 Gheorghe Marinescu Street, 540139 Târgu Mureș, Romania; 3 Laboratory of Antimicrobial Chemotherapy, Ion Ionescu de la Brad University of Life Science, 8 Aleea Mihail Sadoveanu, 700489 Iași, Romania; mihaimares@fungi.ro; 4 Department of General and Inorganic Chemistry, “George Emil Palade” University of Medicine, Pharmacy, Sciences and Technology of Târgu Mureș, 38 Gheorghe Marinescu Street, Mureș, 540139 Târgu Mureș, Romania; lavinia.berta@umfst.ro; 5 Department of Pharmaceutical Botany, “George Emil Palade” University of Medicine, Pharmacy, Sciences and Technology of Târgu Mureș, 38 Gheorghe Marinescu Street, Mureș, 540139 Târgu Mureș, Romania; corneliu.tanase@umfst.ro

**Keywords:** biosynthesis, biofilm, silver nanoparticles, spruce bark, synergism, *SAP2*, *ALS3*, *HSP70*

## Abstract

Due to their high content of biomolecules, combined with silver’s well known antimicrobial potential, silver nanoparticles biosynthesized using spruce bark (AgNP SBEs) demonstrate antibacterial and antioxidant activity, making them a versatile option for developing new antimicrobial agents that might be used for medical treatment or as adjuvants for the classical agents. This study aims to analyze if silver nanoparticles (AgNPs) mediated by spruce bark extract (SBE) and silver salts (AgNP SBE Acetate, AgNP SBE Nitrate) presents antifungal activity against five different *Candida* spp., synergistic activity with fluconazole, and if they interact with some virulence factors of *C. albicans*. AgNP SBEs presented MICs (minimum inhibitory concentrations) for all the five tested *Candida* spp. AgNP SBEs inhibited the growth of *C. parapsilosis*, *C. krusei,* and *C. guilliermondii,* exerted synergistic activity with fluconazole for *C. parapsilosis* and *C. guilliermondii*, and inhibited biofilm production for *C. albicans*, *C. auris*, and *C. guilliermondii*. MICs of AgNP SBE Acetate significantly inhibited the production of germ tubes of *C. albicans*. The expression of *C. albicans* *SAP2* gene was down-regulated by the short-time treatment with MICs of AgNP SBE Acetate, while *ALS3* and *HSP70* genes were up-regulated by the AgNPs MICs. These results emphasize the potential of using the AgNP SBEs as treatments/adjuvants options, not only against the redundant *C. albicans* but also for the non-albicans *Candida* species (which are not as frequently involved in human pathologies, but, sometimes, can be more aggressive).

## 1. Introduction

According to the World Health Organization, infections caused by drug-resistant microorganisms are responsible for at least 700,000 deaths every year, and, if no action will be taken until 2050, this number might increase to 10 million deaths every year [1]. Millions of fungal infections are diagnosed annually, leading to approximately 1,350,000 deaths each year [2]. Since the therapeutic options for these multidrug-resistant microorganisms are very limited, there is an urgent necessity for developing new antimicrobial agents that might be used for treatment or, as adjuvants for the classical agents.

The noble metals such as silver, gold, and platinum, despite the fact that they are considered as non-essential elements for the human organism, are often taken into consideration for several chemical, biological, and medical applications (drug delivery agents, imaging agents, cosmetic applications, antimicrobial medical devices, etc.) due to their antimicrobial, antioxidant, and anti-inflammatory activity [3,4,5,6,7]. In the last few decades, there has been a growing interest in the medical community for different applications of metal-based nanoparticles. Due to their small size, controlled forms, and the possibility of functionalizing their surfaces with other different molecules, the nanoparticles can provide new solutions for solving persistent medical problems, such as the increasing antimicrobial resistance [5,8,9]. Silver nanoparticles appear to be one of the most promising options due to silver’s antimicrobial potential and since the development of bacterial resistance against silver is a very rare phenomenon. It is understood that a bacterial generation needs to undergo three different mutations in three different bacterial systems at the same time to develop resistance against silver, which is unlikely to appear [10,11].

The chemical and physical synthesis of metal-based nanoparticles are considered laborious, expensive, and require toxic reductants (e.g., hydrazine, sodium borohydride) [9,12]. Nanoparticle biosynthesis, especially using different plant extracts, is an interesting option as it is an easy, low-cost method with minimum environmental impact. The plant extracts provide different active agents (such as phenolic, alcoholic compounds, flavones, enzymes, proteins, alkaloids, terpenoids) that facilitate the silver ions reductions [9]. Some of the plant’s biomolecules (flavonoids, phenols) can coat the nanoparticles very effectively, preventing their agglomeration, and by this, the small-sized nanoparticles maintain their ability to penetrate through the bacterial or fungal cell wall [13]. This antimicrobial mechanism, along with the production of reactive oxygen species, membrane alterations, and DNA damages, is already described as antimicrobial mechanism of silver nanoparticles. More detailed studies, with similar methodologies, are necessary to fully understand the antifungal mechanisms of the bio-nanoparticles [11,14,15,16].

*Candida* spp. are considered as the major human fungal pathogens, causing a large variety of infections, from superficial to systemic ones, with the potential of becoming life-threatening infections especially in immunocompromised patients [17,18]. The plasticity and the multiple virulence factors of *Candida* spp. allows them to transform, quickly and easily, from harmless commensals to pathogens. *Candida albicans* adhesion on the host cells and their invasion into the cells is associated with ALS (agglutinin-like sequence) genes [19]. *Als3* is a protein localized on the surface of the hyphae produced by *C. albicans,* that helps it to adhere not only to the host cells but also to the extracellular protein matrix. *C. parapsilosis* presents five ALS genes that are involved in the adhesion to the host cells, while *C. glabrata* presents a different epithelial adhesin (Epa) that promotes this attachment [19,20,21]. *C. albicans* secretes hydrolytic enzymes such as proteinases (Saps), lipases (Lip), phospholipases (PL), which are also involved in the adhesion and the penetration into the host cells. Hsps (heat shock proteins), which mediate the response to stress for *C. albicans*, are involved in the dispersion of these cells inside the biofilms and the susceptibility to antifungal agents [19].

Lately, there is an arising number of studies that focus on the antifungal activity of the silver nanoparticles, and some of their antifungal mechanisms are described. Even if the studies are abundant, most of them are focused especially on *Candida albicans*, as it is the most frequent yeast involved in human infections. However, there is a lack of information regarding the antifungal activity and their antifungal mechanisms against other *Candida* spp., such as the newly discovered multiresistant strain *C. auris,* or *C. krusei*, a long-known drug-resistant strain [2,22,23,24,25].

In previous studies, silver nanoparticles synthesized using spruce bark extract and silver acetate/nitrate were characterized, and their antibacterial, antioxidant activity was described. Since the AgNP SBEs proved to be a versatile antibacterial option for developing new agents with potential to become treatment/adjuvant agents for bacterial infections, we considered important to evaluate if this potential might be extended as an antifungal option, also. [26,27]. This study aims to analyze if these nanoparticles present antifungal activity, against five different *Candida* spp. (*C. albicans*, *C. parapsilosis*, *C. krusei*, *C. auris* and *C. guilliermondii*), if they might exert synergistic activity with different concentrations of fluconazole, and if they might interact with some virulence factors of *C. albicans* (biofilm production, germ tubes formation, the expression of *SAP2*, *ALS3*, and *HSP70* genes).

## 2. Results

### 2.1. Synthesis and Characterization of the AgNP SBEs

Synthesis and characterization of the spruce bark extract (SBE) and AgNP SBEs (AgNP SBE Ac—silver nanoparticles synthesized using spruce bark and silver acetate, AgNP SBE Nit—silver nanoparticles synthesized using spruce bark and silver nitrate) were previously described, in studies conducted by the same collective of authors [26,27]. Briefly, the spruce bark presented a total polyphenol content of 23.67 ± 1.45 mg GAE/g dry weight [26], with compounds as vanillic acid and taxifolin being identified in the SBE [28]. The biosynthesis of the AgNP SBEs was confirmed by the specific color modification and by UV-Vis analysis (maximum absorbance at 411 nm for AgNP SBE Ac, respectively at 431 nm for AgNP SBE Nit). The FTIR analysis of the AgNP SBEs indicated the presence of -O-H bonds (phenolic compounds), -C-H (aldehydes), >C=O, -OH carboxylic, aromatic ethers and carboxylate ions (COO^−^). The transmission electron microscopy images proved that AgNP SBEs were spherical, small size particles (44.02 nm for AgNP SBE Ac, respectively 75.91 nm for AgNP SBE Nit) [26]. As the antibacterial activity of the SBE [28], as well as the antibacterial and antioxidant activity of the AgNP SBEs [26] were previously demonstrated, the next step of our studies was to further investigate (from a microbiological point of view) these AgNPs, by evaluating their antifungal activity against *Candida* spp.

### 2.2. Antifungal Activity of the AgNP Mediated by Spruce Bark Extracts (SBE)

Both the AgNP SBEs presented 50% MICs and 100% MICs for all the five *Candida* spp. (Table 1). Overall, AgNP SBE Nit presented lower MIC values than the AgNP SBE Ac, for both 50% and 100% inhibition. The lowest MIC values were observed for AgNP SBE Nit, in the case of *C. krusei* (0.1 mg/mL for 50% inhibition) and *C. guilliermondii* (0.2 mg/mL for 50% inhibition). For the AgNP SBE Ac, the lowest MICs values were also in the case of *C. krusei* and *C. guilliermondii* (0.045 mg/mL for 50% inhibition). The highest MIC values were registered for C. *auris* and *C. parapsilosis* (AgNP SBE Ac—0.9 mg/mL for 50% inhibition), respectively, for *C. albicans* (AgNP SBE Ac—0.732 mg/mL for 50% inhibition).

### 2.3. The Influence of the AgNP SBEs on the Growth Rate of Candida spp.

**Figure 1:**
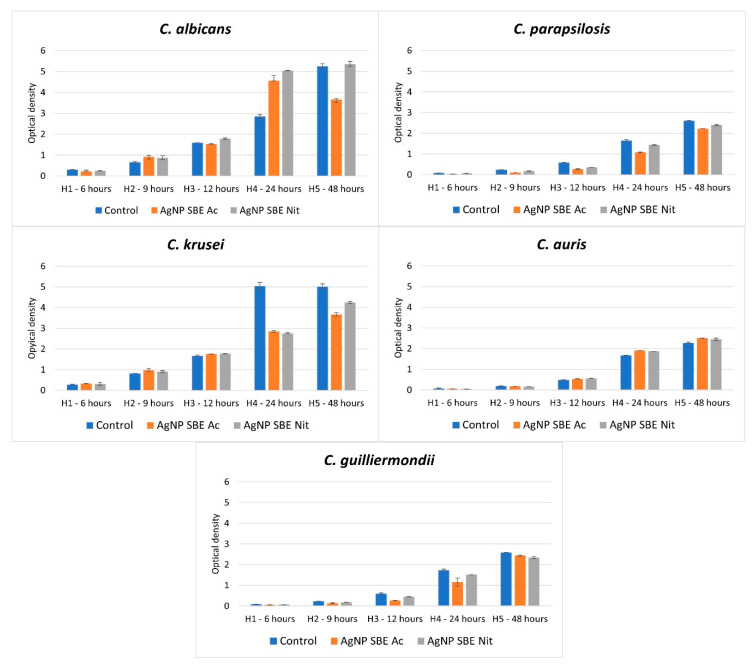
The influence of MICs of AgNP SBEs on the growth rate of *Candida* spp.

After 24 and 48 h of incubation, both AgNP SBEs decreased the growth of *C. parapsilosis*, *C. krusei*, and *C. guilliermondii* (Figure 1). In the case of *C. auris*, the growth rate was not inhibited by MICs of AgNP SBEs. For *C. albicans*, after 24 h of incubation, the growth rate was stimulated by both AgNP SBEs; after 48 h, in the presence of AgNP SBE Nit, the growth rate was almost stationary (compared to the control), while AgNP SBE Ac inhibited the growth rate of *C. albicans*.

### 2.4. Checkerboard Method—Fluconazole Synergy Test

**Figure 2:**
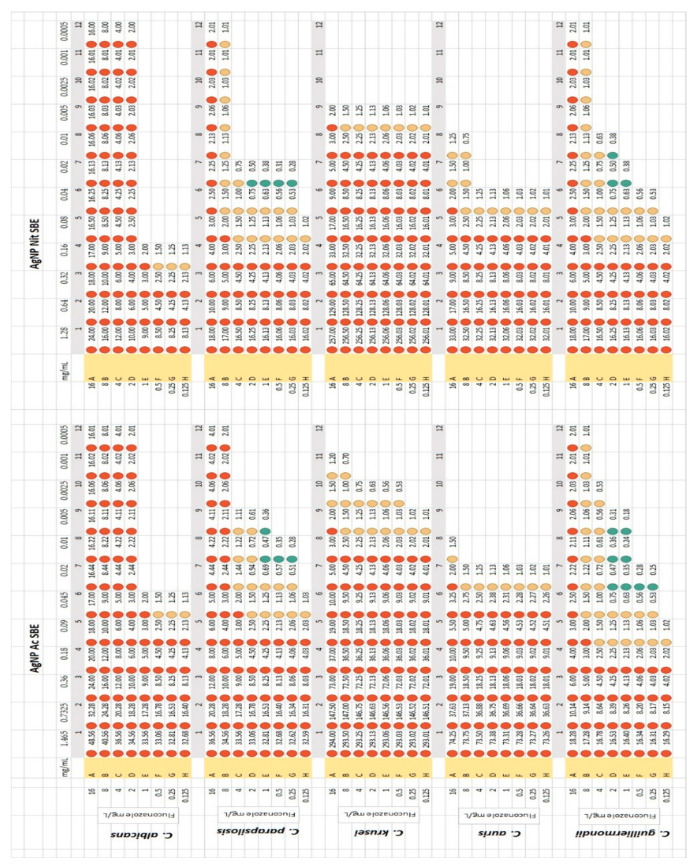
FICs for fluconazole synergy test in the presence of AgNP SBEs (checkerboard method). Synergistic FIC values—marked with green bullets, indifferent FIC values—marked with yellow bullets, antagonistic FIC values—marked with red bullets.

As it is presented in Figure 2, both AgNP SBEs exerted synergistic activity with different concentrations of fluconazole, for *C. parapsilosis*, and *C. guilliermondii*, with the highest number of FIC values (8) registered for *C. guilliermondii*, for different combinations of AgNP SBE Ac (ranging from 0.005 mg/mL to 0.02 mg/mL) and fluconazole (ranging from 0.25 mg/L to 2 mg/L). The lowest FIC values were registered for *C. guilliermondii*, for the combination of AgNP SBE Ac and fluconazole (FIC 0.18 for 0.005 mg/mL AgNP SBE Ac combined with 1 mg/L fluconazole, FIC 0.24 for 0.01 mg/mL AgNP SBE Ac combined with 1 mg/L fluconazole and FIC 0.25 for 0.02 mg/mL AgNP SBE Ac combined with 0.25 mg/L fluconazole). For *C. albicans*, *C. krusei* and *C. auris* no synergistic activity was observed.

### 2.5. The Influence of the AgNP SBEs on the Biofilm Formation

**Figure 3:**
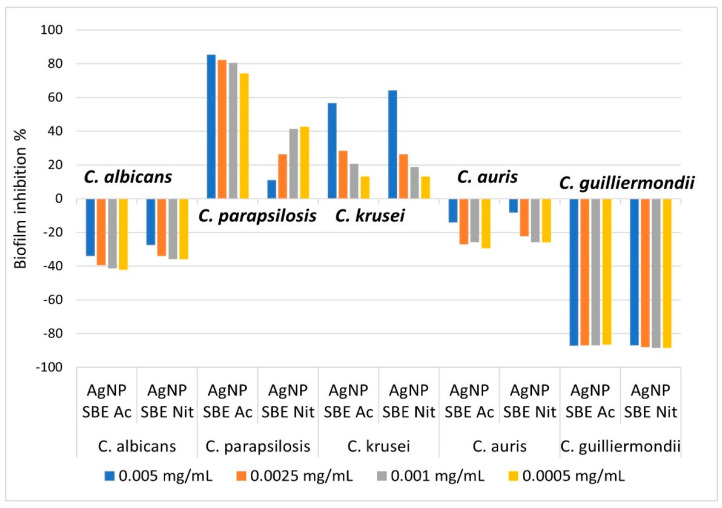
The influence of AgNP SBEs on *Candida* spp. biofilm production (%).

From Figure 3, it can be observed that both the AgNP SBEs inhibited the biofilm production for *C. albicans*, *C. auris* and *C. guilliermondii*. The highest percent of inhibition was observed for *C. guilliermondii* (inhibition percent between 86.63% and 88.47%), followed by *C. albicans* (inhibition percent between 27.52% and 42.2%) and *C. auris* (inhibition percent between 8.23% and 29.41%). The inhibition of the biofilm production was not related to the concentration of AgNP SBEs, as for *C. albicans* and *C. auris* the higher concentrations of AgNP SBEs presented a lower percent of biofilm inhibition than those for the lower concentrations of AgNP SBEs. For *C. guilliermondii*, the percent of biofilm inhibition was almost similar for all the tested AGNP SBEs concentrations. For *C. krusei, C. parapsilosis* AgNP SBEs enhanced the biofilm production on different percentages, as can be seen from Figure 3.

### 2.6. The Influence of the AgNP SBEs on the Germ Tubes Production of C. albicans

**Figure 4:**
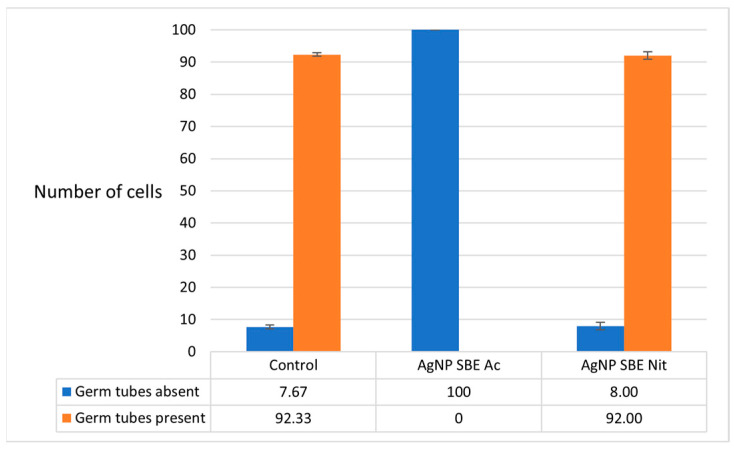
The influence of the AgNP SBEs on the germ tubes production of C. *albicans*.

The MICs of AgNP SBE Ac significantly inhibited the production of germ tubes of *C. albicans* (*p* = 0.0001 compared to both control and AgNP SBE Nit). The production of germ tubes was not influenced by MICs of AgNP SBE Nit (*p* = 1, compared to control) (Figure 4).

### 2.7. The Influence of the AgNP SBEs on C. albicans Gene Expression for ALS3, SAP2, HSP70

**Figure 5:**
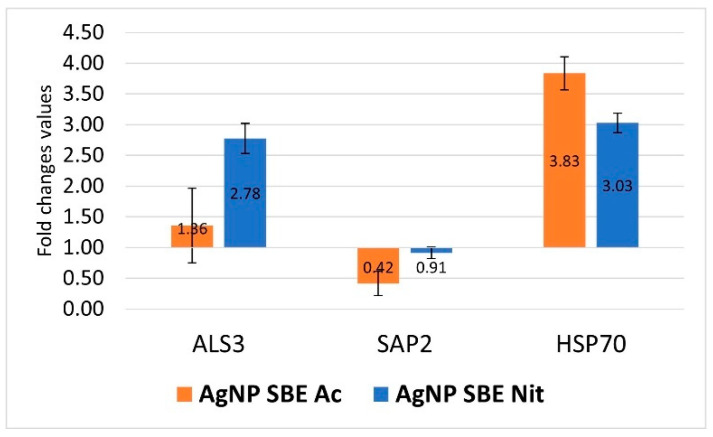
The influence of the AgNP SBEs on *C. albicans* gene expression for *ALS3*, *SAP2*, *HSP70*.

The expression of *C. albicans SAP2* gene was down-regulated by the short time (3 h) treatment with MICs of AgNP SBE Ac (FC = 0.42), while the treatment with AgNP SBE Nit presented an indifferent effect for the *SAP2* expression (FC = 0.91, over the 0.75 value that was considered as the threshold for down-regulation). The expression of *C. albicans ALS3* and *HSP70* gene was up-regulated by both the AgNPs MICs, with smaller FC values for *ALS3* (1.36 for AgNP SBE Ac, respectively, and 2.78 for AgNP SBE Nit) than those obtained for *HSP70* (3.83 for AgNP SBE Ac, respectively, and 3.03 for AgNP SBE Nit) (Figure 5).

## 3. Discussion

It is recognized that the green bio-synthesis methods can generate pure nanoparticles, with controlled morphology and size, free from different toxic contaminants [9,27,29]. Due to their high content of bioactive molecules, different plant extracts not only reduce the silver ions, prevent the agglomeration of the nanoparticles [13], but also brings important antimicrobial, anti-inflammatory, antioxidant, and antitumoral activity, which sums or even enhances the AgNPs activity [30,31,32]. The spruce bark is an interesting option for the biosynthesis of AgNPs due to its availability, high content of gallic, vanillic acids, catechin, astringin, and biomolecules which control the formation of free radicals and presents antibacterial, antioxidant activity [33,34,35,36].

The antibacterial activity of spruce bark and the AgNPs SBEs was previously demonstrated [26,27,28], but their antifungal activity was not described. There are a lot of recent studies that present the antifungal activity of different AgNPs synthesized with different plant extracts against various human pathogens (AgNPs synthesized using Aloe vera leaf extract showed high antifungal activity against *Aspergillus* spp. and *Rhizopus* spp. [37]; AgNPs synthesized with a marine algae, *Hypnea muciformis*, showed antifungal activity against *C. albicans*, *C. parapsilosis*, and *Aspergillus niger* [38]) or against plant pathogens (leaf extract *Ligustrum lucidum* AgNPs demonstrated activity against *Setosphaeria turcica,* turnip leaf extract AgNPs with activity against wood-rotting pathogens [39]). To our knowledge, the antifungal activity of the AgNP SBEs was not studied so far, so our results bring important information for the biological activity of these substances. Both AgNP SBE Ac and AgNP SBE Nit presented MICs for all the studied *Candida* spp., with the lowest values for the AgNP SBE Nit. There are a few antifungal mechanisms already described in the literature such as adherence to the yeast cell membrane, interfering with the fatty acids or ergosterol level, reactive oxygen species production, decreasing membrane fluidity, and DNA alteration [2,40,41]. The small size of the AgNP SBEs [26] and the fact that the bioactive compounds of the plant extracts prevent the agglomeration of the AgNPs [13] might explain their antifungal activity. In our study, the lowest MIC values were observed for AgNP SBE Nit, in the case of *C. krusei* (0.1 mg/mL for 50% inhibition) and *C. guilliermondii* (0.2 mg/mL for 50% inhibition). For the AgNP SBE Ac, the lowest MICs values were also in the case of *C. krusei* and *C. guilliermondii* (0.045 mg/mL for 50% inhibition). These results, along with the AgNP SBEs inhibition of the growth rate for *C. parapsilosis*, *C. krusei* and *C. guilliermondii* emphasizes once again the advantages of using bio-nanoparticles as treatment options, or as adjuvants, even for non-albicans *Candida* species.

Another possible utility of the AgNPs comes from one of their antifungal mechanisms, respectively, that by modifying the fungal membrane permeability, the AgNPs may promote the entry of the fluconazole into the cells wherein it can interfere with the biosynthesis of the ergosterol [42,43]. This might be a possible explanation for our results, as for *C. parapsilosis* and *C. guilliermondii*, both AgNP SBEs exerted synergistic activity with different concentrations of fluconazole, with the highest number of FIC values (8) registered for *C. guilliermondii*, for different combinations of AgNP SBE Ac (ranging from 0.005 mg/mL to 0.02 mg/mL) and fluconazole (ranging from 0.25 mg/L to 2 mg/L). An interesting result was that for *C. krusei* (considered as intrinsically fluconazole-resistant strain), synergistic activity between the fluconazole and the AgNP SBEs was not observed, but for the combination of AgNP SBE Ac with fluconazole, FICs with very close values to the synergy cut-off (0.5) were registered (FIC 0.53 for 0.0025 mg/mL AgNP SBE Ac combined with 0.5 mg/L fluconazole, FIC 0.56 for 0.0025 mg/mL AgNP SBE Ac combined with 1 mg/L fluconazole). Once again, these results draw attention to the possibility of using the AgNP SBEs not only against the redundant *C. albicans*, but, even for the non-albicans *Candida* species, not as frequently involved in human pathologies, but, sometimes, more aggressive.

One of the important virulence factors of *Candida* spp. is their capacity to produces biofilms on different organic and inorganic surfaces. Inside the biofilms, *Candida* spp. cells, protected by the extracellular matrix, are more difficult to eradicate, making these infections a difficult challenge for the physicians, especially when multi-drug resistant strains are involved [19,24,44]. The small size of the green synthesized nanoparticles enables them easy access inside the biofilms matrix, making them a functional option for the inhibition of the biofilms, or as drug delivery agents [45]. Our study demonstrated that both the AgNP SBEs inhibited the biofilm production for *C. albicans*, *C. auris* and *C. guilliermondii*, with the highest percent of inhibition for *C. guilliermondii* (between 86.63% and 88.47%), followed by *C. albicans* (between 27.52% and 42.2%) and *C. auris* (between 8.23% and 29.41%). The inhibition of the *C. auris* biofilm by Ag NPs coated with polyvinylpyrrolidone was demonstrated in another recent study [40].

The morphological transition of *C. albicans* to filamentous forms is another important virulence factor of this yeast, that facilitates its adherence to the host cells, invasion of the cells, and biofilm production [19,23]. Some recent studies reported the *C. albicans* germ tubes inhibition by different AgNPs [23,46,47]. Our study demonstrated that the MICs of AgNP SBE Ac significantly inhibited the production of germ tubes of *C. albicans*, but the production of germ tubes was not influenced by MICs of AgNP SBE Nit. The Ras-mediated signal transduction pathways might be affected by biosynthesized AgNPs, by down-regulating the expression of the genes involved in the morphological transition of C. *albicans* (*EGE1*—cell elongation gene; *TEC* gene—hyphal inducer gene; *TUP1*, *RFG1*—hyphal transition genes) [23,46,48].

*C. albicans* secretes hydrolytic enzymes such as proteinases (Saps), lipases (Lip), and phospholipases (PL), which are involved in the adhesion and the penetration into the host cells. Hsps, (heat shock proteins), which mediate the response to stress for *C. albicans*, are involved in the dispersion of these cells inside the biofilms and the susceptibility to antifungal agents [19]. The up/down regulation of the expression of different genes might increase/decrease the virulence of the fungal strain by stimulating/inhibiting different virulence factors, encoded by the gene. Our study showed that the expression of *C. albicans SAP2* gene was down-regulated by the short time (3 h) treatment with MICs of AgNP SBE Ac, while the *ALS3* and *HSP70* genes were up-regulated by both the AgNPs MICs. ALS (agglutinin-like sequence) genes are associated with *Candida albicans* adhesion on the host cells and their invasion into the cells. *Als3* is a protein localized on *C. albicans* hyphae that helps the yeast to attach to the cells and, also, to the extracellular protein matrix [19]. Even if both the AgNP SBEs inhibited the biofilm formation, and the MICs of AgNP SBE Ac significantly inhibited the production of germ tubes of *C. albicans*, the expression of *C. albicans ALS3* and *HSP70* gene (genes that are both involved in the biofilm formation) was up-regulated by both the AgNPs MICs. These results emphasize the possibility that multiple other mechanisms of actions are involved in the antifungal activity of biosynthesized AgNPs.

The results from this study and previously published ones [26,27,28,36] sustain the possibility of AgNP SBEs functionalization as antifungal and antibacterial agents, but more research is needed to fully understand their antimicrobial mechanisms and how these AgNPs might be used for different medical applications.

## 4. Materials and Methods

### 4.1. The Synthesis of the Silver Nanoparticles

The spruce bark (*Picea abies* L.) aqueous extract was obtained from spruce bark waste, according to previously published studies [26,28]. The silver nanoparticles (AgNPs) were synthesized and characterized following a previously published protocol [26,27]. The obtained nanoparticles solutions were silver nanoparticles produced with silver acetate and spruce bark (AgNP SBE Ac), 2.93 mg AgNPs/mL, and silver nanoparticles produced with silver nitrate and spruce bark (AgNP SBE Nit), 2.56 mg AgNPs/mL. The obtained solutions were filtered using sterile syringe filters, with 0.45 µm diameter, to ensure that the work solutions were sterile.

### 4.2. Fungal Strains

Five *Candida* spp. were used in this study: *C. albicans* ATCC 90028, *Candida parapsilosis* ATCC 22019, *C. krusei* ATCC 6258, *C. auris* CBS 10913, *C. guilliermondii* 184 (Cantacuzino National Research and Development Institute for Microbiology and Immunology, Bucharest). The strains are stored at −70 °C in the Microbiology Department from “George Emil Palade” University of Medicine, Pharmacy, Science, and Technology of Târgu Mureş and at the Laboratory of Antimicrobial Chemotherapy, Ion Ionescu de la Brad University of Life Sciences, Iași. *Candida* spp. were revitalized before each experiment in Sabouraud Dextrose Broth (SDB, Basingstoke, Oxoid, UK).

### 4.3. Antifungal Activity of the AgNP SBEs

For the assessment of the antifungal activity of the AgNP SBEs, we performed the microdilution method recommended by EUCAST [49], with minor modification. Briefly, from 200 µL of AgNP SBEs, serial two folded dilutions were performed in RPMI 2X medium (Sigma Aldrich, St. Louis, MO, USA) buffered with MOPS, in the rows of a 96 wells microtiter plate. An inoculum with approximatively 1–5 × 10^5^ CFU/mL density was prepared, by mixing 9 mL RPMI with 1 mL 0.5 McFarland fungal suspension. From this inoculum, 100 µL were pipetted to the microtiter-plate’s wells. A positive well (fungal inoculum + RPMI) and a negative one (RPMI medium) were performed as controls. After 36 h of incubation, at 37 °C, the 50% minimum inhibitory concentration (MIC) was evaluated by visual examination, in the wells where the *Candida* spp. growth was over 50% inhibited, comparing to the control well. The 100% MIC was considered in the wells without visible growth. Tests were performed in triplicate.

### 4.4. The Influence of the AgNP SBEs on the Growth Rate of Candida spp.

The influence of the AgNP SBEs on the growth rate of *Candida* spp. was performed in 10 mL volume suspensions that reproduced the MICs for each tested substance. The tubes were incubated at 37 °C, for 48 h. As growth control, we used a tube with the same volume and fungal inoculum, but without AgNPs SBEs. From these suspensions, 400 µL were removed at 0, 6, 9, 12, 24, 48 h. Optical density (OD) was assessed at 600 nm, using a spectrophotometer (BioPhotometer D30, Eppendorf, Wien, Austria). Tests were done in triplicate.

### 4.5. Checkerboard Method—Fluconazole Synergy Test

For each *Candida* spp. and tested AgNP SBEs, the checkerboard method [31] was performed, to evaluate if the AgNP SBEs might exert synergistic activity with different concentrations of fluconazole. The plates were designed to include (on the same plate) the clinical breakpoints of the tested *Candida* spp. (were available, according to EUCAST recommendations) [49]. From the first wells of the horizontal axis of the microtiter plates, 200 µL of AgNP SBEs were serial binary diluted in 100 µL distilled water. From a fluconazole (Sigma Aldrich, St. Louis, MO, USA) stock solution of 132 mg/L, two folded dilutions were prepared in RPMI 2X, and 50 µL from each of these dilutions were added on all the wells from each row of the vertical axis of the microtiter plates (the final concentration of the fluconazole in the first row was 16 mg/L, while in the last row was 0.125 mg/L). Fifty microliters of 0.5 McFarland fungal inoculum (1:10 diluted in RPMI 2X), were added in all the wells of the microtiter plates. The positive and negative controls were considered the wells from opposite corners of the microtiter plate, where the concentration of both fluconazole and AgNP SBEs were highest, respectively, lowest. After the plates were incubated at 37 °C, for 48 h, the MICs were assessed at over 50% growth inhibition (by visual examination), and the fractional inhibitory concentration was calculated for all the wells where the fungal growth was inhibited over 50%, using the following formula:

$$FIC=\frac{fluconazole\;MIC\;from\;the\;studied\;well}{fluconazole\;MIC}+\frac{AgNP\;SBE\;MIC\;from\;the\;studied\;well}{AgNP\;SBE\;MIC}$$

FIC values ≤ 0.5 were interpreted as synergic effect of the tested solutions with fluconazole; FIC values between 0.5–2, were considered as an indifferent effect; if FIC values were > 2, the effect of the tested solutions was considered antagonistic [50].

### 4.6. The Influence of the AgNP SBEs on the Biofilm Production

The influence of the AgNP SBEs on the production of biofilms was evaluated using the crystal violet staining method [51], in the MIC microtiter plates, after the MICs were assessed. The last four columns of the microtiter plates were used for evaluating the biofilm inhibition, as these were the columns in which all the *Candida* spp. presented full growth, for all the tested *Candida* strains, and the AgNP SBEs (colored solutions) were diluted enough not to influence the optical density of the well’s suspensions at the spectrophotometric evaluation. After the excess RPMI medium was removed, the wells of the microtiter plates were immersed twice in sterile water, to wash away the unattached cells. Afterward, 200 µL of 0.1% aqueous crystal violet solution were pipetted in each well, and the plates were incubated for 15 min, at room temperature. After the crystal violet solution was removed, the plates were immersed three times in sterile water and kept at room temperature to evaporate the water excess. The crystal violet that remained attached to the cells was solubilized with 200 µL of 30% acid acetic (room temperature, 15 min). The suspensions from the wells were homogenized by gently shaking the plates, and each well’s absorbance was measured (at 620 nm, using a spectrophotometer). The positive control well was used as a control for the influence of the biofilm formation, as this well did not contain the AgNP SPEs. The biofilm inhibition was assessed using the next formula:

$$\%\;of\;biofilm\;inhibition=\left(100*\frac{absorbance\;value\;of\;the\;assessed\;well}{absorbance\;value\;of\;the\;control\;well}\right)-100$$

### 4.7. The Influence of the AgNP SBEs on the Germ Tubes Production of C. albicans

To evaluate how the AgNP SBE influence the *C. albicans* germ tubes production, MICs of the AgNP SBEs were replicated in a 0.5 mL serum volume and homogenized with the same volume of *C. albicans* suspension (0.5 McFarland). For the germ tubes production control, 0.5 mL serum was mixed with 0.5 mL fungal suspension. The tubes were incubated at 36 °C, for 2 h, in a water bath. The number of the germ tubes/100 cells was assessed by microscopically examining wet mounts (prepared from 25 µL of the incubated suspensions). Tests were performed in triplicate.

### 4.8. The Influence of the AgNP SBEs on C. albicans Gene Expression for ALS3, SAP2, HSP70

*C. albicans* was revitalized in SDB, by incubating it overnight at 37 °C, in an orbital shaker. One milliliter of 2.1 AU (OD measured at 600 nm-BioPhotometer D30, Eppendorf, Austria, Wien) was centrifuged at 5000 rpm for 5 min; the obtained sediment was suspended in 1.5 mL of AgNP SBEs MICs prepared with SDB, and incubated for three hours, in a thermomixer, at 37 °C.

After the incubation, the AgNP SBEs treated samples were centrifugated 5 min at 5000 rpm, and the sediment was resuspended in 500 µL TE buffer. Two cycles of freezing and heating (−70 °C, respectively, and 95 °C) were performed to damage the fungal cell wall, followed by the digestion of the cell wall with *Arthrobacter luteus* lyticase (Sigma Aldrich, St. Louis, MO, USA). Afterward, the lyticase was neutralized by heating the samples for 10 min at 94 °C, and silica-based spin columns were used for RNA purification. The unwanted DNA from the elutes was removed using DNase I solution (Thermo Scientific, Waltham, MA, USA). After the purity and concentration of the obtained RNA (ng/µL) were assessed by nanodrop reading (Eppendorf BioPhotometer D30, Wien, Austria), reverse-transcription was carried out (GoScript Reverse Transcription System, Promega, Madison, WI, USA) to produce complementary DNA.

QuantStudio 5 Real-Time PCR System (Thermo Fisher, Waltham, MA, USA) was used to perform the RT-PCR assay in a final volume of 20 µL. The following primers were used: for *ACT1*—forward 5′–TTG TTG ACC GAA GCT CCA ATG–3′ and reverse 5′–ACG TGA GTA ACA CCA TCA CCA–3′; for *ALS3*—forward 5′–CCA CTT CAC AAT CCC CAT C–3′ and reverse 5′–CAG CAG TAG TAG TAA CAG TAG TAG TTT CAT C–3′; for *SAP2*—forward 5′–GAT GCT GCC ACG GGA CAA AT–3′ and reverse 5′–AGA AGC AGC AAA TTC GGA AGC–3′; for *HSP70*—forward 5′–TGG TAT TCC ACC AGC TCC AAG–3′ and reverse 5′–CAA CTT CTT CAA CAG TTG GTC CAC–3′). Afterward, the *ALS3*, *SAP2*, *HSP70* expression levels were adjusted by reporting to the *ACT1* levels, using the Delt-Delta Ct Method [52]. Fold changes (FC) values ˂ 0.75 were interpreted as down-regulation, while FC > 1.25, were considered up-regulation. Tests were done in triplicate.

## 5. Conclusions

Both the AgNP SBEs presented different degrees of inhibitions for all the tested *Candida* spp, with an overall better activity for the AgNP SBE Nit. The growth rate of *C. parapsilosis*, *C. krusei,* and *C. guilliermondii* was inhibited by both the AgNP SBEs.

Both AgNP SBEs exerted synergistic activity with different concentrations of fluconazole for *C. parapsilosis* and *C. guilliermondii*, drawing attention to the possibility of functionalization these nanoparticles as adjuvants for the classical antifungal agents.

Both the AgNP SBEs inhibited the biofilm production for *C. albicans*, *C. auris* and *C. guilliermondii*, with a higher percent of inhibition for *C. guilliermondii*, followed by *C. albicans* and *C. auris*. MICs of AgNP SBE Ac significantly inhibited the production of germ tubes of *C. albicans*, but the production of germ tubes was not influenced by MICs of AgNP SBE Nit. The expression of *C. albicans SAP2* gene was down-regulated by the short-time treatment with MICs of AgNP SBE Ac, while *ALS3* and *HSP70* gene were up-regulated by both the AgNPs MICs. Our results conclude that these nanoparticles might be an option as modulating agents for the different virulence factors of *Candida* spp.

The overall antifungal activity of the AgNP SBEs emphasizes the possibility of using them as treatment/adjuvant options, not only against the redundant *C. albicans*, but, even for the non-albicans *Candida* species, not as frequently involved in human pathologies, but sometimes more aggressive.

## Figures and Tables

**Table 1:** Antifungal activity (MIC) of AgNP SBEs for *Candida* spp.

*Candida* spp.	50% Inhibition		100% Inhibition	
	AgNP SBE Ac mg/mL	AgNP SBE Nit mg/mL	AgNP SBE Ac mg/mL	AgNP SBE Nit mg/mL
*C. albicans*	0.732	0.16	1.465	0.64
*C. parapsilosis*	0.9	0.04	0.36	0.32
*C. krusei*	0.045	0.01	0.09	0.02
*C. auris*	0.9	0.08	0.18	0.16
*C. guilliermondii*	0.045	0.02	0.09	0.08
